# How to culturally adapt mental mHealth apps: lessons from an Australian Arabic-speaking community survey

**DOI:** 10.3389/fdgth.2026.1801244

**Published:** 2026-05-19

**Authors:** Noorah Ibrahim S. Alnaghaimshi, Michael Proeve, Scott R. Clark, Mathias Baumert

**Affiliations:** 1 Discipline of Biomedical Engineering, School of Electrical and Mechanical Engineering, Adelaide University, Adelaide, SA, Australia; 2 Department of Computer and Information Science, Majmaah University, Al-Majmaah, Riyadh Region, Saudi Arabia; 3 School of Psychology, Faculty of Health and Medical Sciences, Adelaide University, Adelaide, SA, Australia; 4 Discipline of Psychiatry, School of Medicine, Adelaide University, Adelaide, SA, Australia; 5 Department of Technical Physics, University of Eastern Finland, Kuopio, Finland

**Keywords:** Arabic migrants, cultural adaptation, mental health, mHealth, religion

## Abstract

**Background:**

Culturally and religiously responsive mental mobile health (mHealth) apps may improve access to and acceptability of mental health support among migrant communities; however, evidence to inform their design remains limited.

**Objective:**

This formative study investigated mental health perceptions, digital health information-seeking, and mental mHealth app use among first-generation Arabic-speaking migrants in Australia, with the aim of informing culturally adapted mental mHealth app design.

**Methods:**

An online survey was conducted among 219 first-generation Arabic-speaking migrants in Australia (aged 18–75 years), recruited from non-clinical community settings. The survey assessed attitudes toward mental health, awareness and use of mental mHealth apps, acceptance of app-based support, and desired features. Open-ended questions provided qualitative insights into cultural and religious preferences.

**Results:**

Strong cultural and religious influences on mental health perceptions were observed, including high agreement regarding the role of divine will and religious practices. While most participants (76.3%) used the internet to seek mental health information, awareness (45.7%) and use (6.4%) of mental mHealth apps were low. Participants expressed high acceptance of mental mHealth apps that are free, user-friendly, confidential, and professionally developed. Highly valued features included culturally informed behavioural activation, mindfulness and religious practices (such as *Dua’a* and *Tadabbur*), and educational content incorporating Quranic verses and prophetic narratives. Information on crisis services and local multicultural mental health providers was also considered essential. Qualitative findings supported the inclusion of faith-based community features and religious motivational content, with several participants emphasising the importance of optional rather than mandatory religious elements.

**Conclusions:**

There is a clear demand for mental mHealth apps tailored to the cultural and religious needs of first-generation Arabic-speaking migrants in Australia. Formative evidence from this study highlights the importance of culturally and religiously congruent design, practical support features, confidentiality, and flexibility to accommodate individual preferences when developing mental mHealth interventions.

## 1 Introduction

Mobile health (mHealth) apps offer a scalable solution for delivering self-help, educational content, and interventions for mental disorders, thereby facilitating self-management (1). They can effectively reduce symptoms of depression and anxiety, with those delivering Cognitive Behavioural Therapy (CBT) showing the strongest evidence (2). mHealth apps are particularly valuable for providing mental health and well-being resources in a cost-effective, private, and non-stigmatizing format, and potentially personalised (3). This makes them especially beneficial for underserved populations, including those in racial and ethnic minorities, rural and remote communities, financially deprived groups, and refugee and migrant communities (3).

One such community that could significantly benefit from these digital health solutions is Arabic-speaking imigrants, many of whom face unique mental health challenges, having arrived as refugees or displaced persons from conflict-affected regions like Iraq and Syria (4, 5). This demographic group faces significant mental health challenges, experiencing higher rates of psychological distress and trauma exposure, increasing their risk of developing PTSD and major depressive disorder (6). These issues are often exacerbated by pre-migration trauma, post-displacement stressors, and insecure visa status (7–10). Despite these known challenges, data gaps exist regarding the prevalence of mental disorders, their determinants, and the provision of services for immigrant and refugee populations (11).

A critical need exists for culturally adapted mental healthcare for Arabs, as current services face numerous barriers to access and engagement (6). Deep-seated cultural and religious beliefs often attribute mental illness to external factors such as satanic powers or lack of faith, rather than psychological issues (12), leading to significant shame and stigma and prompting concealment and a reluctance to seek formal mental health support (12). The strong emphasis on family honour means a mental health diagnosis can negatively reflect on the entire family, further encouraging secrecy (12, 13). Consequently, trust in formal services is low, compounded by language barriers, insufficient culturally tailored promotion, and confidentiality concerns (12). Many prefer seeking help from religious leaders as their initial point of contact (12). Therefore, integrating religious principles, such as “Amal Saleh” (righteous deeds), into mental health interventions could be beneficial for enhancement of acceptability and engagement. Such practices align with users' existing spiritual coping mechanisms and cultural values, directly addressing the aforementioned barriers to conventional mental healthcare access.

Technology, particularly mental mHealth apps, offers a promising avenue for anonymous and flexible care, potentially overcoming social stigma and increasing access (14, 15). Addressing the needs of specific demographics requires strategies that circumvent stigma and align with cultural values. Delivering interventions in non-stigmatizing settings is crucial (16). Furthermore, given the collective identity and family-centric nature of Arab culture, actively involving families and ensuring their support is vital for effective therapeutic outcomes (12, 17). Indeed, a study on culturally adapted mindfulness programs for Arabic-speaking migrants showed high levels of cultural acceptability and significant improvement in mental health outcomes, demonstrating how selected interventions can be successfully tailored to cultural and religious practices (18).

In this study we aimed to identify critical design considerations for mHealth delivery of CBT tailored to Arabic-speaking individuals experiencing depression to enhance uptake, user engagement and benefit. We developed an e-survey to: (1) assess the mental health landscape among Arabic-speaking immigrants in Australia; (2) examine the awareness and knowledge of digital mental health resources and prior engagement with such resources (websites and applications); (3) explore the influence of cultural and religious beliefs on mental health perceptions and treatment-seeking among Arabic immigrants; (4) assess attitudes towards mHealth smartphone applications; (5) identify factors that would encourage or discourage participants from using mental health apps; and (6) ascertain the perceived importance of various features and tools for a culturally and religiously sensitive mental health app.

## 2 Methods

### 2.1 E-survey design

We developed an electronic survey instrument comprising 72 items, integrating multiple-choice questions, Likert scales, and free-text answers. The scope of the questionnaire encompassed demographic information, a brief mental health history checklist, including recent symptoms (19, 20), questions on cultural and religious influences on mental health (21–23), and assessments of attitudes towards, and barriers to, e-health and m-health interventions, alongside preferences for app features (24–29).

To ensure linguistic and cultural appropriateness for the target population, the survey was developed in both English and Arabic versions (Supplementary Materials S1 and S2). The English version of the survey was translated into Arabic by the author NA, a native speaker. Subsequently, both the original English and the translated Arabic versions were independently reviewed by two academic native speakers to assess the translation's validity and reliability. Following this, the survey was deployed via the REDCap online platform. A pilot study involving four participants from the target group was conducted to evaluate the clarity and functionality of the e-survey. Feedback garnered from this pilot phase informed necessary refinements, culminating in the final version of the e-survey for broader dissemination. Participants were given the option to complete the survey in Arabic or English, depending on their linguistic proficiency.

### 2.2 Recruitment procedures

We recruited participants from non-clinical settings, leveraging community groups and various institutional and social platforms, including universities, mosques, community language schools, and Arabic Facebook and WhatsApp groups, primarily through flyer distribution. Collaborative partnerships with several organizations, namely Survivors of Torture and Trauma Assistance and Rehabilitation Service (STTARS), Multicultural Communities Council of South Australia (MCCSA), and Australian Migrant Resource Centre (AMRC), facilitated the identification of prospective participants and the dissemination of study information through their established communication channels and client networks.

### 2.3 Inclusion criteria

Survey participants were included if they were aged 18 to 75, currently lived in Australia, qualified as a “first-generation Arabic migrant” (i.e., individuals born overseas), were born in one of 22 Arabic countries, and used Arabic as their first language.

### 2.4 Data analysis

Prior to analysis, all optionally provided personally identifiable information was removed from participant data. Participants' qualitative inputs were coded (e.g., P1, P2, P3). Quantitative data analysis was conducted using IBM SPSS Statistics 31. Frequencies and percentages were used to describe demographic characteristics, while means and standard deviations summarized response patterns. Cronbach's alpha was employed to test internal consistency reliability, and correlation coefficients were used to assess construct validity. Thematic analysis (TA) was applied to qualitative data derived from open-ended responses using the AI tool MAXQDA 24. Inductive thematic analysis, following Braun and Clarke (30), was conducted by NA on Arabic data. English coding was line by line, with memoing and iterative clustering to identify emergent themes, and interpretive translation was conducted as needed.

## 3 Results

### 3.1 Instrument validation and reliability testing

This section details the validity of the study's questionnaire categories and its instrument reliability. Construct validity was confirmed by assessing the correlation between each construct and the total questionnaire score. As presented in Supplementary Materials S3, all constructs exhibited significant correlations (*p* < 0.05), with coefficients ranging from 0.230 to 0.907. Supplementary Table S3 also shows a high Cronbach's alpha of 0.925 for the entire questionnaire. Individual category values ranged from 0.677 (Cultural and religious influences) to 0.948 (Useful mHealth app features), indicating validity and reliability for the target population.

### 3.2 Participant characteristics

Of the 338 survey responses, 226 were complete. After excluding seven ineligible cases that did not meet the inclusion criteria, 219 responses were retained for analysis. The majority of participants were female (82.6%), and almost half were aged 35–44 years, whereas only 4.1% were aged 55–64 years (Table 1). Most respondents were married (79.5%), and 42% held a bachelor's degree. Only 0.9% reported having no formal education. About one-third identified as stay-at-home parents. Egypt was the most common country of birth (26.9%), followed by Syria (19.6%), while 32.9% of participants were born outside the regions listed in the study instrument (Supplementary Material S4).

**Table 1:** Demographic characteristics of the 219 participants.

Variable	Category	Frequency (*n*)	Percentage (%)
Gender	Male	38	17.4
	Female	181	82.6
Age group (years)	18–24	14	6.4
	25–34	54	24.7
	35–44	102	46.6
	45–54	40	18.3
	55–64	9	4.1
Marital status	Single, never married	21	9.6
	In a relation	1	0.5
	Engaged	2	0.9
	Married	174	79.5
	Divorced	13	5.9
	Widowed	8	3.7
Education	No schooling completed	2	0.9
	Basic/compulsory education (primary – preparatory)	10	4.6
	High school or equivalent	30	13.7
	Diploma (two to three years after high school)	18	8.2
	Bachelor's degree	92	42.0
	Postgraduate degree	67	30.6
Employment status	Student	34	15.5
	Employed	82	37.4
	Self-employed	17	7.8
	Stay-at-home mum/dad	64	29.2
	Unemployed	18	8.2
	Retired	4	1.8
Country of birth	Egypt	59	26.9
	Syria	43	19.6
	Iraq	20	9.1
	Palestine	9	4.1
	Lebanon	16	7.3
	Other (see Supplementary Materials S4)	72	32.9
Current Australian immigration status	Naturalized citizen (citizenship by conferral)	119	54.3
	Permanent resident	61	27.9
	Refugee	9	4.1
	Asylum seeker	8	3.7
	Other	22	10.0
Number of years lived in Australia	Less than 2 years	38	17.4
	2–4 years	33	15.1
	5–7 years	50	22.8
	8–10 years	36	16.4
	More than 10 years	62	28.3

### 3.3 Mental health and well-being profile of participants

About 38.8% of participants reported having been diagnosed with depression, and 22.4% had previously received individual psychotherapy (e.g., CBT). Only 11.9% had participated in group psychotherapy sessions (e.g., CBT, support, or psycho-educational groups).

Beyond these clinical indicators, the research also explored participants' broader subjective experiences of mental health and well-being, encompassing dimensions such as positive attitude, comfort, satisfaction, and interpersonal relationships (Table 2). The study sample exhibited a moderate overall level of mental health and well-being. For example, while participants frequently reported positive experiences such as “enjoying time with family or friends” with an average of 3.90 out of 5.00 and a mean of 78.0%, they also indicated experiencing “tiredness or low energy” with an average of 2.59 out of 5.00 and a mean of 51.8%, and “low feelings, or mood swings” with an average of 2.71 out of 5.00 and a mean of 54.2% some of the time.

**Table 2:** Participants’ broader subjective experiences of mental health and well-being.

No.	Item	Mean	Percent	Std. Deviation	Agreement Level	Rank
1	Have you experienced a positive attitude towards your life?	3.68	73.6%	0.882	Most of the time	2
2	Have you felt comfortable, serene, or peaceful?	3.58	71.6%	0.866	Most of the time	3
3	Have you experienced tiredness or low energy?	2.59	51.8%	0.881	Most of the time	8
4	Have you experienced low feelings, or mood swings?	2.71	54.2%	0.907	Some of the time	7
5	Have you experienced satisfaction from what you do?	3.57	71.4%	0.877	Most of the time	4
6	Have you experienced a positive attitude towards your interpersonal relationships?	3.49	69.8%	0.935	Most of the time	5
7	Have you enjoyed spending time with family or friends?	3.90	78.0%	1.053	Most of the time	1
8	Have you felt that you've let yourself or others down?	3.26	65.2%	1.133	Some of the time	6

### 3.4 Awareness, use, and experience with digital mental health tools

The majority (76.3%) of participants reported using the internet for mental health information, indicating a general propensity for online health advice seeking. However, awareness of dedicated therapeutic mental health websites and apps was considerably lower (45.7%), and actual usage was minimal (6.4%), highlighting a substantial gap between general online information seeking and engagement with digital therapeutic tools.

Qualitative survey feedback on the limited use and short-term engagement with mental m-Health apps attributed this to “No time” or reliance on “free features”. These practical barriers, such as time constraints and cost, align with the quantitative findings of low uptake. A substantial portion of qualitative feedback emphasised the need for incorporating cultural and religious elements into mental m-Health apps to enhance adoption and engagement.

### 3.5 Perceived influence of culture and religion on mental health

Participants were asked about the extent of the influence of culture and religion on mental health. As shown in Table 3, the highest level of agreement was observed for the statement, “Everything happens only by the will and destiny of God”, which yielded a mean of 4.66 (93.2%), categorised as *Strongly Agree*. This was closely followed by the statement “Following religious practices strengthens the psychological health of the individual”, with a mean of 4.56 (91.2%) and a rating of *Strongly Agree*. Additionally, “Performing various acts of worship, such as prayer, and reading the Holy Book or Scripture, is a path to healing” received strong agreement with a mean of 4.49 (89.8%).

**Table 3:** Extent of influence of culture and religion on mental health.

No.	Item	Mean	Percent	Std. Deviation	Agreement Level	Rank
1	Everything happens only by the will and destiny of God.	4.66	93.2	0.769	Strongly Agree	1
2	Following religious practices strengthens the psychological health of the individual.	4.56	91.2	0.760	Strongly Agree	2
3	Performing various acts of worship, such as prayer, and reading the Holy Book or Scripture, is a path to healing.	4.49	89.8	0.885	Strongly Agree	3
4	I would prefer to turn to my religion rather than engaging with mental health treatment.	3.65	73.0	1.266	Strongly Agree	4
5	Anyone experiencing mental illness should keep it hidden, even from those who are closest, to avoid the stigma.	1.99	39.8	1.139	Disagree	6
6	I would require family consent and support for seeking and continuing mental health therapy.	3.06	61.2	1.348	Uncertain	5

Conversely, the lowest level of agreement was for the statement, “Anyone experiencing mental illness should keep it hidden, even from those who are closest, to avoid the stigma”, which had a mean of 1.99 (39.8%). The majority of respondents were uncertain regarding the statement “I would require family consent and support for seeking and continuing mental health therapy”, showing a mean of 3.06 (61.2%).

### 3.6 Attitude towards mHealth apps

Participants' intention to use mHealth apps for specific functions such as finding information, relieving emotional problems, online counseling, and self-management (Table 4) is likely influenced by a lack of prior experience, as detailed in section 3.4, suggesting that limited awareness and direct experience likely contribute to a more neutral rather than strongly positive general attitude towards these tools. However, when the survey questions shifted to specific features, particularly those incorporating cultural and religious adaptations, as detailed in section 3.8, the perceived importance of these features was significantly higher, often rated “Very important”. This contrast suggests that while general exposure to/use of mHealth apps are low, integrating culturally and religiously relevant features could significantly enhance their perceived value and potential acceptance among users.

**Table 4:** Participants’ intention to use mHealth apps.

No.	Item	Mean	Percent	Std. Deviation	Agreement Level	Rank
1	I would use an app to find information about mental health problems/concerns.	2.81	56.2%	1.062	Sometimes	1
2	I would use an app to relieve or assist me with my emotional problem(s).	2.57	51.4%	1.070	Rarely	4
3	I would use an app to get online counselling from a psychiatrist/psychologist/psychotherapist/counsellor.	2.67	53.4%	1.205	Sometimes	3
4	I would use an app that helps me self-manage my mental health.	2.71	54.2%	1.206	Sometimes	2

### 3.7 Factors influencing acceptance and barriers to mHealth app adoption

Participants demonstrated a high degree of positive sentiment regarding factors associated with app quality, safety and accessibility (Table 5). The most significant factor encouraging the use of mHealth apps was the app being free, closely followed by “App is simple and effortless to use”. Other highly encouraging factors included the app's development with input from specialists and health professionals, its provision of relevant and reliable information, and its customizable functionalities. Slightly less important were the availability of the native language (Arabic) and the use of data encryption measures.

**Table 5:** Participant perspectives on mHealth app safety, quality and accessibility.

No.	Item	Mean	Percent	Std. Deviation	Agreement Level	Rank
1	My native language (Arabic) is an option in the app.	4.06	81.2	0.873	Encourage use	11
2	App's privacy policy explains how data is handled and stored.	4.13	82.6	0.870	Encourage use	10
3	App uses data encryption measures.	3.95	79.0	0.908	Encourage use	12
4	App enables me to get help and support while maintaining complete confidentiality of my identity.	4.21	84.2	0.847	Strongly encourage use	8
5	App and its content were developed with the input of specialists and health professionals.	4.29	85.8	0.811	Strongly encourage use	3
6	App comes from an authorized/official entity (e.g., developed by government, hospital, university).	4.27	85.4	0.793	Strongly encourage use	6
7	App supplies a self-assessment or management tool(s) that are medical or scientifically approved.	4.16	83.2	0.819	Encourage use	9
8	App provides relevant and reliable information.	4.29	85.8	0.826	Strongly encourage use	3 rep.
9	App aids access to professional care and other mental health resources.	4.24	84.8	0.771	Strongly encourage use	7
10	The app is free.	4.45	89.0	0.761	Strongly encourage use	1
11	App is simple and effortless to use (i.e., usable).	4.44	88.8	0.807	Strongly encourage use	2
12	Apps have customizable functionalities that allow users to tailor some app features to their needs and preferences.	4.29	85.8	0.798	Strongly encourage use	3 rep.

### 3.8 User-rated features of CBT for mHealth apps

mHealth app features designed for CBT intervention were categorized into five key areas: behavioural activation, mindfulness/reflection/coping/relaxation, educational/motivational content, user's self-help journey, and support. Overall, these features were rated as *Very important* (mean=3.71, 74.2%) (Table 6).

**Table 6:** Summary of importance ratings for mHealth app feature categories.

No.	Category	Mean	Percent	Std. Deviation	Agreement Level	Rank
1	Behavioural activation/righteous deeds/community activities	3.90	78.0	0.828	Very important	1
2	Mindfulness/reflection/coping/relaxation	3.85	77.0	0.943	Very important	2
3	Educational/motivational content	3.84	76.8	.955	Very important	3
4	User's self-help journey	3.75	75.0	0.778	Very important	4
5	Support	3.38	67.6	0.915	Important	5
Total		3.71	74.2	0.‏714	Very important	

#### 3.8.1 Behavioural activation/righteous deeds/community activities

This category was identified as the most important overall, with a mean rating of 3.90 (78.0%). The means for items within this category ranged from 3.85 to 3.95 out of 5.00 (Supplementary Material S5). The highest-ranked feature in this category was “Incorporating activities that reflect some of the collectivistic values (such as attending family gatherings, visiting neighbours, participating in community events)”. This feature achieved a mean of 3.95 (79.0%) and was rated *very important.* This was followed by “Incorporating both behavioural activation, an effective approach in CBT, a secular-based therapeutic model and some of the Islamic religious principles of Amal Saleh “righteous deeds” (such as acts of worship, charity and helping others)”, a mean of 3.91 (78.2%).

Qualitative responses strongly reinforced the value of community and culturally relevant activities. Participants expressed a desire for features that foster social connection, particularly within a shared cultural or religious context. For example, Participant (P. 46) suggested “Faith-Based Group Chats and Support Circles: Enable community or group chat options that allow people to connect over shared cultural or religious identities”. This highlights that the high quantitative rating for collectivistic values stems from a desire for communal support that resonates with users' cultural and religious backgrounds.

#### 3.8.2 Mindfulness/reflection/coping/relaxation

This category also received a high overall importance rating, with a mean of 3.85 (77.0%), indicating it is very important for enhancing mindfulness and relaxation. Item means ranged from 3.56 to 4.14 out of 5.00 (Supplementary Material S6). The most highly valued feature was “Incorporating Imtinān/Shukr of Allah ‘gratitude’ as a mindfulness exercise or coping tool”, with a mean of 4.14 (82.8%). Other Islamic practices, such as ‘Incorporating Dua’a 'supplication’’ and “Incorporating Tadabbur” (reflection on the Al-Qur'an), were also rated as *very important*.

Qualitative responses provided deeper insight into why these specific features are considered important. Participant (P. 67), reflecting on personal experience, emphasized the profound impact of religious doctrines:

I immensely encourage incorporating religious doctrines in the app, particularly the Quran.. if it wasn't for Quran and all the beautiful motivational phrases… and gives you examples of people like prophets that struggled with hardships for years and eventually levitated out of this hardships and mental agony to a more peaceful and amicable life.. I was sad, afraid, anxious, hopeless and physically idle.. after I constantly prayed and read Quran.. suddenly, out of the blue, you start seeing light at the end of the tunnel.

This narrative illustrates the perceived efficacy of religious practices in coping and achieving emotional balance, directly supporting the high quantitative ratings for features such as gratitude, supplication, and Quranic reflection. Participant (P. 49) further detailed specific practices, including *Ruqyah*, *Istighfar*, and reading or listening to *Surah Al-Baqarah*, providing concrete examples of desired mindfulness and coping tools.

#### 3.8.3 Educational/motivational content

This category was rated as *Very important* overall, with a mean of 3.84 (76.8%). The means for items in this category ranged from 3.27 to 4.16 out of 5.00 (Supplementary Material S7).The most critical feature identified was “Incorporating verses from the Quran and Hadith that remind one of relief after struggle, reward with patience, and encourage acceptance, resilience, and hope”, achieving a mean of 4.16 (83.2%) and was rated as *very important.* This was followed by “Incorporating narratives of prophets who have suffered hardship and trauma (such as Prophet Ayyub and Prophet Musa)” with a mean of 4.11 (82.2%).

Qualitative data strongly corroborated the demand for religiously grounded motivational content. Participant (P.42) explicitly suggested “incorporating verses from the Quran and Sunnah” to “instill hope in the heart of anyone suffering from psychological problems”. This directly underscores the quantitative importance of Quranic and Hadith verses, highlighting their role in providing comfort, resilience, and hope during mental health struggles. The qualitative data thus provide the rationale for the strong preference for such content. While many participants strongly advocated for religiously grounded motivational content, Participant (P.275) suggested keeping “this religiously related content optional based on the person's preferences”, acknowledging its potential irrelevance for non-religious users. This highlights the need for personalized app experiences.

#### 3.8.4 User's self-help journey

The overall mean for the “User's self-help journey” category was 3.75 (75.0%), indicating a *very important* aspect for respondents. Item means ranged from 3.67 to 3.89 out of 5.00 (Table 7). The highest-ranked feature was “Progress tracking: Allows user to track progress and achieve milestones”. This feature had a mean of 3.89 (77.8%) and was rated *very important*. This was closely followed by 'Standalone features: Self-help tools (e.g., for performing tasbeeh, breathing) for coping with sad moments or distressing thoughts, accessible anytime, and includes interactive components such as a timer' with a mean of 3.78 (75.6%).

**Table 7:** Participant ratings for user self-help journey features.

No.	Item	Mean	Percent	Std. Deviation	Agreement Level	Rank
1	Progressive engagement path: A structured, step-by-step daily programme that may be used at any time of day and that gradually introduces educational content and corresponding behavioural activation and mindfulness tasks and exercises.	3.74	74.8	0.894	Very important	4
2	Gamified learning: Tasks and exercises introduced into the daily programme, such as challenges with rewards for completion.	3.67	73.4	0.925	Very important	6
3	Scheduled reminders: Allows user to schedule reminders for daily programmes at times that suit their routines.	3.72	74.4	0.954	Very important	5
4	Contextual reminders: Automated reminders that alert user based on progress in completing daily programmes following periods of absence.	3.67	73.4	0.935	Very important	6 rep.
5	Self-paced learning: Allow user to access a supplementary educational content and engage with it in own time without being restricted by the daily programme structure.	3.77	75.4	0.954	Very important	3
6	Standalone features: Self-help tools (e.g., for performing tasbeeh, breathing) for coping with sad moments or distressing thoughts, accessible anytime, and includes interactive components such as a timer.	3.78	75.6	0.996	Very important	2
7	Progress tracking: Allows user to track progress and achieve milestones.	3.89	77.8	0.949	Very important	1

The qualitative data strongly implied a “progress tracking” value, though participants did not explicitly mention the theme of overcoming adversity through spiritual and self-help engagement. Participant (P.67), detailing clarity and advancement through consistent prayer and Quran reading, as a means of dealing with adversity, exemplifies the importance of tracking progress. Consequently, this qualitative insight suggests that users value a clear trajectory and the ability to monitor their progress in self-help efforts, thereby reinforcing the high quantitative rating for “Progress tracking”. Furthermore, the high importance of “Standalone features,” which include tools like Tasbeeh, resonates with qualitative data advocating for the integration of specific religious practices for coping and mindfulness, as suggested by participant (P.99) for various Islamic remembrances. This indicates a strong preference for accessible, on-demand tools that are culturally and religiously congruent, enabling users to manage distress effectively as part of their personal self-help journey.

#### 3.8.5 Support

This category was rated as *Important* overall by respondents, with a mean of 3.38 (67.6%). The means for items in this category ranged from 3.09 to 3.84 out of 5.00 (Table 8). Within this category, “Information on crisis response services that are available 24/7, and that explain how to obtain an interpreter to assist in communicating with any of those services” was the most important feature, with a mean of 3.84 (76.8%). This was followed by “Information on relevant national and local providers and mental health services for multicultural communities in Australia,” which received a mean score of 3.66 (73.2%). Qualitative data strongly corroborated the demand for access to professional and crisis support. Participants explicitly requested features such as “contact with doctors, a mental health professional, with a symbolic or even free fee” (P.58) and “adding free psychological treatment access” (P.282). This confirms user demand for direct, professional intervention and readily available crisis resources.

**Table 8:** Participant ratings for support features.

No.	Item	Mean	Percent	Std. Deviation	Agreement Level	Rank
1	Option to add or remove a family member or friend to engage in app to provide support and encouragement.	3.24	64.8	1.212	Important	4
2	Deliver information and educational material about related mental health concerns and issues to the selected family member or friend (supporters) in order to reduce stigma and boost their supportive capacities.	3.32	66.4	1.212	Important	3
3	Option to share specific aspects of personal progress or achievements with the selected family member or friend (supporters) (e.g., completed tasks).	3.14	62.8	1.190	Important	5
4	A messaging or commenting feature that allows selected family members or friends (supporters) to send supportive messages, comments, and feedback.	3.09	61.8	1.218	Important	6
5	Information on relevant national and local providers and mental health services for multicultural communities in Australia.	3.66	73.2	0.998	Very Important	2
6	Information on crisis response services that are available 24/7, and that explains how to obtain an interpreter to assist in communicating with any of those services.	3.84	76.8	1.009	Very Important	1

While features enabling family and friend support, such as “Option to add or remove a family member or friend to engage in app to provide support and encouragement” and “Deliver information and educational material about related mental health concerns and issues to the selected family member or friend (supporters) to reduce stigma and boost their supportive capacities”, received a mean of 3.24 (64.8%) and 3.32 (66.4%) respectively, and were both rated as *Important*. These scores were somewhat lower than those for crisis information. However, the qualitative data strongly suggest their importance within the cultural context. As highlighted in section 3.8.1, the emphasis on collectivistic values and the desire for the feature “Faith-Based Group Chats and Support Circles” indicate that support from close social networks, including family, is culturally significant. Participant (P.51) explicitly suggested a feature for “Family group chat and video conferencing”, further underscoring the perceived utility of integrating family into the support system. These findings demonstrate that such features which include trusted friends and family are suitable and important for the target cultural context, aligning with existing social structures and values that prioritize family and community involvement in mental health support. This suggests that the design of features related to providing "support" requires careful consideration to integrate both professional and culturally-relevant social networks effectively.

## 4 Limitations of the study

Our study sample comprised a mix of people with and without self-reported depression (38.8% diagnosed with depression, 61.2% undiagnosed), represents a diverse range of opinions rather than a narrow perspective from clinically diagnosed patients.Recruitment from community settings may have introduced selection bias, potentially overrepresenting those who were more digitally literate or socially connected. While demographic factors such as gender, age, and marital status may have influenced participants' responses and preferences, a detailed analysis of these factors' specific influences was beyond the study scope of this descriptive, formative study and would require a balanced sampling strategy (the majority of participants were female and married).

Social desirability bias when addressing sensitive topics like religion and cultural values may have affected our findings. Our use of an online survey format, which offers a degree of anonymity that encourages more candid responses, mitigates this issue to some degree. Qualitative feedback from participants, highlighted the importance of optional rather than mandatory religious elements suggesting a thoughtful engagement with the survey questions beyond mere social conformity.

We aimed to engage a broad spectrum of first-generation Arabic-speaking migrants in Australia through diverse community platforms, including universities, mosques, community language schools, and Arabic social media groups. To avoid introducing additional sampling bias we did not explicitly collect data on participants' religious affiliations and survey items on cultural and religious influence were intentionally phrased to be inclusive of various faiths. In this context the qualitative and quantitative feedback on desired religious features overwhelmingly highlighted specifically Islamic practices and texts (e.g., Quranic verses, Hadith, Dua'a, Tadabbur, Tasbeeh), suggesting that the majority of participants who provided input on religious content were likely Muslim, which aligns with the high prevalence of Islam in the Middle East, North Africa (MENA) region (31), Consequently, our findings might not be generalisability beyond the Arab’ community.

A key finding is the low prior experience with therapeutic digital applications and online mental health programs among participants, with only 6.4% reporting previous use. This limited exposure, possibly due to unfamiliarity, awareness (32) or availability of such apps in Arabic-speaking contexts (33), means participant preferences reflected initial perceptions rather than opinions refined by extensive usage. Low digital literacy is a factor that Fan et al. (34)identify as a strong impact on mHealth app adoption. While acknowledging its importance, a detailed investigation into the specific role of digital literacy was beyond the current scope of this study. Nevertheless, a “fresh perspective” is valuable for identifying initial adoption barriers and desired features for new or culturally adapted mental health applications.

The pre-categorization of “Useful mHealth App features” into researcher-defined themes (e.g., “Behavioural activation/righteous deeds/community activities”, “Mindfulness/reflection/coping/relaxation”) represents a methodological limitation. This framework, though logical for an initial assessment, may have inadvertently constrained the discovery of other valuable or innovative features not included in the predefined categories, potentially limiting the comprehensive understanding of users' unprompted needs and priorities for app functionalities. To overcome this, co-design sessions were conducted in the subsequent research phase to explore user needs more deeply and generate new ideas, the results of which will be presented in a separate paper.

## 5 Discussion and conclusion

The data from our survey strongly underscore the critical need for cultural and religious adaptation in digital mental health interventions, particularly self-help mHealth apps. While there is a general awareness of mental health websites and apps, and a significant portion of respondents use the internet to seek mental health information, actual engagement with therapeutic digital apps remains low. Only a small percentage (6.4%) reported having tried such apps, with the vast majority indicating they had not. This low uptake and short adoption suggest that existing apps may not be sufficiently attractive or meet the specific needs of this population (35), highlighting a potential disconnect between available resources and user needs, which culturally and religiously sensitive designs could address. Furthermore, varying levels of digital literacy may also contribute to this low engagement (36). This aligns with findings from a recent systematic review (37), which emphasise the importance of culturally responsive self-care strategies for Arabic-speaking refugees and migrants. This minimal engagement, despite a general openness to digital health solutions, strongly implies that the current offerings fail to resonate with the cultural and religious frameworks integral to this population's understanding of well-being and coping mechanisms. Additionally, unfamiliarity with digital interventions in mental health can impact usage (32).

A key finding is the high level of cultural and religious influence among study participants. Respondents strongly agreed that “Everything happens only by the will and destiny of God” and that “Following religious practices strengthens the psychological health of the individual”. This indicates that for a significant user base, spirituality and faith are integral to their understanding of well-being and coping mechanisms. The preference to “turn to my religion rather than engaging with mental health treatment” further emphasizes this point, suggesting that traditional mental health approaches may not fully resonate with individuals holding strong religious beliefs. This aligns with previous studies (34, 38) that underscore how, in many Arab communities, cultural and religious frameworks not only influence how mental distress is interpreted but also steer people towards spiritual, religious coping and away from professional mental health interventions. This pattern must be acknowledged when considering mental-health policy, outreach, and culturally responsive care, making this preference for religious practices over formal mental health interventions a crucial consideration for developers of digital interventions. However, it is equally crucial to address instances where religious beliefs can contribute to negative coping mechanisms, such as attributing mental illness to satanic powers or lack of faith (39). Negative coping mechanisms are associated with increased anxiety, worry, and depression, highlighting the importance of religiously sensitive psychoeducation within mHealth apps (39). Content should harmonize seeking medical help with religious faith, thereby challenging the view of mental illness as a spiritual failure and mitigating the harmful effects of negative religious attributions.

Interestingly, the lowest level of agreement was recorded for the statement, “Anyone experiencing mental illness should keep it hidden, even from those who are closest, to avoid the stigma”. On the experience of stigma Chimoriya et al. (40) reported high levels of mental health stigma across Arab communities in Australia, and Slewa-Younan et al. (41) found that Arabic-speaking women experience higher personal stigma than men. This could be attributed to participants' long exposure to Australian culture, their high level of educational levels, or other contextual influences that may reduce stigma expression. However, this intriguing finding warrants further investigation to clarify the relationships and fully understand the factors contributing to lower observed stigma in this population, including acculturation and other contextual influences on attitudes toward mental health. Despite this, the influence of cultural and religious beliefs still impacts participant's desire to access help, as indicated by the low percentage of who had been diagnosed with depression (only 22.4%) and who engaged in individual or group psychotherapy (11.9%). The gap between lower self-reported stigma and low treatment rates may be attributed to systemic barriers, such as financial costs, language barriers, lack of culturally responsive providers, and general distrust in formal services, as extensively documented by Khatib et al. (34).

Features incorporating collectivistic values and Islamic religious principles, such as Amal Saleh (righteous deeds), were rated as *very important*. This directly underscores the need for Cultural CBT adaptation (CA-CBT), integrating therapeutic strategies with community-oriented values and practices (42). Furthermore, the importance of religion-adapted CBT (R-CBT) (43) is evident in the high value placed on features such as incorporating gratitude (Imtinān/Shukr of Allah), supplication (Dua'a), and reflection on the Quran (Tadabbur) as mindfulness or coping tools. Additionally, incorporating verses from the Quran and Hadith that encourage acceptance, resilience, and hope for educational/motivational content. These elements are not merely supplementary; they are considered very important for enhancing mindfulness and relaxation. Qualitative feedback further supported these findings, indicating a strong preference for religious content among those who identify as religious, although it has been suggested that it should be optional.

In conclusion, for mHealth self-help apps to be effective and widely adopted within culturally and religiously diverse populations, a generic, one-size-fits-all approach is insufficient. The influence of culture and religion cannot be ignored (34, 44). The data unequivocally demonstrate a strong preference and perceived importance for interventions that are deeply integrated with users' cultural and religious frameworks. Therefore, the development of *digital* mental health tools for Arabs must prioritize religious CBT adaptation (denoted as R-iCBT), by incorporating spiritual practices, religious texts, and faith-based coping mechanisms, and cultural CBT adaptation (denoted as CA-iCBT), by integrating collectivistic values and community-oriented activities (i.e., R-iCBT+ CA-iCBT) (33), while also considering strategies to enhance digital literacy and awareness of the availability of digital interventions among target users. Such adaptations are not just about adding features; they are about creating interventions that resonate authentically with users' views, thereby fostering greater engagement, acceptance, and ultimately, more effective digital mental health intervention and support. This also necessitates a clear strategy for maintaining user confidentiality and privacy, particularly when incorporating family or community features. The design must prioritise user control, ensuring that sharing with family or community is entirely optional and that privacy policies for all data are transparently outlined. This includes making religious content optional, recognising that while highly valued by many, it may not be suitable or desired by all users, including those who may have experienced religious trauma or hold secular views. This approach directly embodies the flexibility to accommodate essential individual preferences, allowing users to tailor their engagement with social and religious elements to their personal comfort and privacy needs.

## Data Availability

The original contributions presented in the study are included in the article/Supplementary Material, further inquiries can be directed to the corresponding author.

## References

[1] Miralles I Granell C Díaz-Sanahuja L Van Woensel W Bretón-López J Mira A Smartphone apps for the treatment of mental disorders: systematic review. JMIR Mhealth Uhealth. (2020) 8(4):e14897. 10.2196/14897 32238332 PMC7163422

[2] Linardon J Torous J Firth J Cuijpers P Messer M Fuller-Tyszkiewicz M. Current evidence on the efficacy of mental health smartphone apps for symptoms of depression and anxiety. A meta-analysis of 176 randomized controlled trials. World Psychiatry. (2024) 23(1):139–49. 10.1002/wps.21183 38214614 PMC10785982

[3] Bear HA Ayala Nunes L Ramos G Manchanda T Fernandes B Chabursky S The acceptability, engagement, and feasibility of mental health apps for marginalized and underserved young people: systematic review and qualitative study. J Med Internet Res. (2024) 26:e48964. 10.2196/48964 39078699 PMC11322694

[4] Australian Red Cross. Refugee and asylum seeker facts (2023). Available online at: https://www.redcross.org.au/act/help-refugees/refugee-facts/https://www.redcross.org.au/act/help-refugees/refugee-facts/ (Accessed August 30, 2024).

[5] Australian Bureau of Statistics. Census Country of birth QuickStats (2021). Available online at: https://www.abs.gov.au/census/find-census-data/quickstats/2021/4206_AUS (Accessed August 30, 2024).

[6] Slewa-Younan S Mond JM Bussion E Melkonian M Mohammad Y Dover H Psychological trauma and help seeking behaviour amongst resettled Iraqi refugees in attending English tuition classes in Australia. Int J Ment Health Syst. (2015) 9:1–6. 10.1186/1752-4458-9-5 25972917 PMC4429497

[7] Chen W Hall BJ Ling L Renzaho AMN. Pre-migration and post-migration factors associated with mental health in humanitarian migrants in Australia and the moderation effect of post-migration stressors: findings from the first wave data of the BNLA cohort study. Lancet Psychiatry. (2017) 4(3):218–29. 10.1016/S2215-0366(17)30032-9 28161455

[8] Schweitzer RD Brough M Vromans L Asic-Kobe M. Mental health of newly arrived Burmese refugees in Australia: contributions of pre-migration and post-migration experience. Aust N Z J Psychiatry. (2011) 45(4):299–307. 10.3109/00048674.2010.543412 21303193

[9] Schweitzer R Melville F Steel Z Lacherez P. Trauma, post-migration living difficulties, and social support as predictors of psychological adjustment in resettled Sudanese refugees. Aust N Z J Psychiatry. (2006) 40(2):179–87. 10.1080/j.1440-1614.2006.01766.x 16476137

[10] Nickerson A Byrow Y O’Donnell M Mau V McMahon T Pajak R The association between visa insecurity and mental health, disability and social engagement in refugees living in Australia. Eur J Psychotraumatol. (2019) 10(1):1688129. 10.1080/20008198.2019.1688129 32002133 PMC6968544

[11] Minas H Kakuma R Too L Vayani H Orapeleng S Prasad-Ildes R Mental health research and evaluation in multicultural Australia: developing a culture of inclusion. Int J Ment Health Syst. (2013) 7:1–25. 10.1186/1752-4458-7-23 24093216 PMC3852843

[12] Youssef J Deane FP. Factors influencing mental-health help-seeking in Arabic-speaking communities in Sydney, Australia. Ment Health Relig Cult. (2006) 9(1):43–66. 10.1080/13674670512331335686

[13] Alhomaizi D Alsaidi S Moalie A Muradwij N Borba CPC Lincoln AK. An exploration of the help-seeking behaviors of Arab-Muslims in the US: a socio-ecological approach. J Muslim Ment Health. (2018) 12(1):19–46. 10.3998/jmmh.10381607.0012.102

[14] Doraiswamy PM London E Varnum P Harvey B Saxena S Tottman S Empowering 8 billion minds: enabling better mental health for all via the ethical adoption of technologies. NAM Perspect. (2019) 2019. 10.31478/201910b 34532674 PMC8406599

[15] Naslund JA Deng D. Addressing mental health stigma in low-income and middle-income countries: a new frontier for digital mental health. Ethics Med Public Health. (2021) 19:100719. 10.1016/j.jemep.2021.100719 35083375 PMC8786211

[16] Al-Krenawi A Graham JR Dean YZ Eltaiba N. Cross-national study of attitudes towards seeking professional help: Jordan, United Arab Emirates (UAE) and Arabs in Israel. Int J Soc Psychiatry. (2004) 50(2):102–14. 10.1177/0020764004040957 15293428

[17] Almoshmosh N Jefee Bahloul H Barkil-Oteo A Hassan G Kirmayer LJ. Mental health of resettled Syrian refugees: a practical cross-cultural guide for practitioners. J Mental Health Train Educ Pract. (2020) 15(1):20–32. 10.1108/JMHTEP-03-2019-0013

[18] Blignault I Saab H Woodland L Mannan H Kaur A. Effectiveness of a community-based group mindfulness program tailored for Arabic and Bangla-speaking migrants. Int J Ment Health Syst. (2021) 15(1):32. 10.1186/s13033-021-00456-0 33849610 PMC8042358

[19] Al-Shehri AH Taha AZ Bahnassy AA Salah M. Health-related quality of life in type 2 diabetic patients. Ann Saudi Med. (2008) 28(5):352–60. 10.5144/0256-4947.2008.352 18779640 PMC6074492

[20] Sam DL Eide R. Survey of mental health of foreign students. Scand J Psychol. (1991) 32(1):22–30. 10.1111/j.1467-9450.1991.tb00849.x 2047794

[21] Thomas J Al-Qarni N Furber SW. Conceptualising mental health in the United Arab Emirates: the perspective of traditional healers. Ment Health Relig Cult. (2015) 18(2):134–45. 10.1080/13674676.2015.1010196

[22] Al-Darmaki F Thomas J Yaaqeib S. Mental health beliefs amongst Emirati female college students. Community Ment Health J. (2016) 52:233–8. 10.1007/s10597-015-9918-9 26286081

[23] Jalal B Samir SW Hinton DE. Adaptation of CBT for traumatized Egyptians: examples from culturally adapted CBT (CA-CBT). Cogn Behav Pract. (2017) 24(1):58–71. 10.1016/j.cbpra.2016.03.001

[24] Kamel MM Westenberg JN Choi F Tabi K Badawy A Ramy H Electronic mental health as an option for Egyptian psychiatry: cross-sectional study. JMIR Ment Health. (2020) 7(8):e19591. 10.2196/19591 32788155 PMC7453323

[25] Krebs P Duncan DT. Health app use among US mobile phone owners: a national survey. JMIR Mhealth Uhealth. (2015) 3(4):e4924. 10.2196/mhealth.4924 PMC4704953 26537656

[26] Mamdouh M Tai AMY Westenberg JN Shams F Jang K Badawy A Egyptian students open to digital mental health care: cross-sectional survey. JMIR Form Res. (2022) 6(3):e31727. 10.2196/31727 35311692 PMC8981018

[27] Kebede MM Pischke CR. Popular diabetes apps and the impact of diabetes app use on self-care behaviour: a survey among the digital community of persons with diabetes on social media. Front Endocrinol (Lausanne). (2019) 10:135. 10.3389/fendo.2019.00135 30881349 PMC6407478

[28] Torous J Wisniewski H Liu G Keshavan M. Mental health mobile phone app usage, concerns, and benefits among psychiatric outpatients: comparative survey study. JMIR Ment Health. (2018) 5(4):e11715. 10.2196/11715 30446484 PMC6269625

[29] Atallah N Khalifa M El Metwally A Househ M. The prevalence and usage of mobile health applications among mental health patients in Saudi Arabia. Comput Methods Programs Biomed. (2018) 156:163–8. 10.1016/j.cmpb.2017.12.002 29428068

[30] Braun V Clarke V. Using thematic analysis in psychology. Qual Res Psychol. (2006) 3(2):77–101. 10.1191/1478088706qp063oa

[31] Alkhaibari RA Smith-Merry J Forsyth R Raymundo GM. Patient-centered care in the Middle East and north African region: a systematic literature review. BMC Health Serv Res. (2023) 23(1):135. 10.1186/s12913-023-09132-0 36759898 PMC9909864

[32] Ftanou M Machlin A Mangelsdorf SN Morgan A Bassilios B. Australian digital mental health services: consumer perceptions of their usability and acceptability. J Technol Behav Sci. (2025) 10(2):320–35. 10.1007/s41347-024-00431-9

[33] Alnaghaimshi NIS Awadalla MS Clark SR Baumert M. A systematic review of features and content quality of Arabic mental mHealth apps. Front Digit Health. (2024) 6:1472251. 10.3389/fdgth.2024.1472251 39723151 PMC11668747

[34] Khatib HE Alyafei A Shaikh M. Understanding experiences of mental health help-seeking in Arab populations around the world: a systematic review and narrative synthesis. BMC psychiatry. (2023) 23(1):324. 10.1186/s12888-023-04827-4 37161342 PMC10170733

[35] Ashfaq A Esmaili S Najjar M Batool F Mukatash T Al-Ani HA Utilization of mobile mental health services among Syrian refugees and other vulnerable Arab populations—a systematic review. Int J Environ Res Public Health. (2020) 17(4):1295. 10.3390/ijerph17041295 32085422 PMC7068284

[36] Fan S Jain RC Kankanhalli MS. A comprehensive picture of factors affecting user willingness to use mobile health applications. ACM Trans Comput Healthcare. (2024) 5(1):1–31. 10.1145/3626962

[37] Mehjabeen D Blignault I Taha PH Reavley N Slewa-Younan S. A mixed methods systematic review of mental health self-care strategies for Arabic-speaking refugees and migrants. BMC public Health. (2023) 23(1):2544. 10.1186/s12889-023-17395-9 38124024 PMC10731719

[38] Al Shelali M Alibrahim H Alomar N Pandi-Perumal SR Seeman MV Jahrami H. The good, the bad, and the ugly of faith healers and psychiatric illnesses: a systematic review of the literature in the Arab world. J Relig Health. (2024) 63(2):857–76. 10.1007/s10943-023-01898-1 37626227

[39] O'Brien B Shrestha S Stanley MA Pargament KI Cummings J Kunik ME Positive and negative religious coping as predictors of distress among minority older adults. Int J Geriatr Psychiatry. (2019) 34(1):54–9. 10.1002/gps.4983 30375027

[40] Chimoriya R Mohammad Y Thomson R Webster C Dunne R Aibangbee M Mental illness stigma and associated factors among Arabic-speaking refugee and migrant populations in Australia. Int J Ment Health Syst. (2023) 17(1):11. 10.1186/s13033-023-00580-z 37138317 PMC10155307

[41] Slewa-Younan S Narchal R Das R Krstanoska-Blazeska K Blignault I Li B Gender, mental health stigma, and help-seeking in Arabic-and Swahili-speaking communities in Australia. Int J Environ Res Public Health. (2024) 21(12):1619. 10.3390/ijerph21121619 39767460 PMC11675820

[42] Huey SJ Jr Park AL Galán CA Wang CX. Culturally responsive cognitive behavioral therapy for ethnically diverse populations. Annu Rev Clin Psychol (2023) 19: 51–78. 10.1146/annurev-clinpsy-080921-072750 36854287

[43] de Abreu Costa M Moreira-Almeida A. Religion-adapted cognitive behavioral therapy: a review and description of techniques. J Relig Health. (2022) 61(1):443–66. 10.1007/s10943-021-01345-z 34518980 PMC8837510

[44] Alqasir A Ohtsuka K. The impact of religio-cultural beliefs and superstitions in shaping the understanding of mental disorders and mental health treatment among Arab Muslims. J Spiritual Ment Health. (2024) 26(3):279–302. 10.1080/19349637.2023.2224778

