# Supplementary material for: How to culturally adapt mental mHealth apps: lessons from an Australian Arabic-speaking community survey
Source: Front Digit Health. 2026 May 19;8:1801244. doi: 10.3389/fdgth.2026.1801244 (PMC13226535; doi:10.3389/fdgth.2026.1801244)
Supplement: Supplementary file 1 [file Datasheet1.docx]

Supplementary Material

# Supplementary Data

## Supplemental Material S1. English version of the survey

**Questionnaire to assess Arabic immigrants’ use of mobile applications for improving**

**mental health**

This survey aims to develop an understanding of how and to what extent Arab immigrant communities in Australia use mental health applications and factors that may influence such use.

**Survey Instructions**

This questionnaire includes statements on a variety of topics. Please read these statements and their choices before selecting only one of the available options. It is important to note that there is no right or wrong answer; you must give your thoughts on these statements. Your participation in the study is entirely voluntary, and withdrawing from it will have no consequences. Your answers will be kept confidential, and deidentified data will be used to ensure anonymity. The findings will only be used for research purposes.

Before you begin the survey, please note that some questions may be sensitive in nature. Your well-being is important to us. If at any point you feel distressed, please contact with your GP, counsellor, or please refer to the resources listed below.

| **Service name** | **Description** | **Phone number** | **Web address** |
| --- | --- | --- | --- |
| Beyond Blue | Offers free and confidential online and telephone helpline services for individuals dealing with depression, anxiety, or other mental health issues, as well as those seeking to support a loved one. You can call them 24 hours a day, 7 days a week. | 1300 22 4636 | [https://www.beyondblue.org.au/home](https://umhcc.org.au/) |
| Lifeline | Offers free support for individuals experiencing a personal crisis or have suicidal thoughts. You can call them 24 hours a day, 7 days a week. | 13 11 14 | <https://www.lifeline.org.au> |
| Urgent Mental Health Care Centre (UMHCC) | A free service, open 24 hours a day, 7 days a week. offers an alternative to presenting at hospital Emergency Departments for a mental health crisis. | 0884489100 | [https://umhcc.org.au/](https://www.migrantwomensservices.com.au/domestic-violince-service/) |
| Migrant womens services | Provides free support to non-English speaking women and children from diverse backgrounds, including Arabic speakers. Offers trauma-informed counseling and therapy to aid in healing from domestic violence. | ------------------ | [https://www.migrantwomensservices.com.au/domestic-violince-service/](tel:1300%20727%20957) |
| Survivors of Torture and Trauma Assistance and Rehabilitation Service (STTARS) | Offer free counselling and support services for refugees and migrants who have experienced torture or trauma. | (08) 8206 8900 | <https://www.sttars.org.au/about/about-sttars> |
| Lives Lived Well | Offer mental health and well-being supports services for families and children, including culturally and linguistically diverse backgrounds communities | [1300 727 957](https://umhcc.org.au/) | [https://www.liveslivedwell.org.au/our-services/mental-health/northern-adelaide/](https://www.redcross.org.au/act/help-refugees/refugee-facts/https://www.redcross.org.au/act/help-refugees/refugee-facts/) |
| Embrace Multicultural Mental Health | Offer accessible resources translated into languages including Arabic, and links to services | ------------------ | [https://embracementalhealth.org.au/community/multilingual-information](https://www.migrantwomensservices.com.au/domestic-violince-service/) |

**Terminology**

For this survey:

- “Mental health” means the ability to cope with regular crises while maintaining a positive sense of fulfilment and adequacy, usually attained by meeting the essential needs of security, love, self-affirmation, achievement, and success.
- “Mental health disorders / Mental illness” means health conditions involving changes in the person’s cognition, emotional control, mood, thinking, perceptions, or behaviour (or a mix of these).
- “Mental health app” is a smartphone app that is primarily directed to help those in need by giving tools to assess, prevent, support, and manage different aspects of mental health.
  1. Demographic information:

| - Gender: | - Male | - Female |  |
| --- | --- | --- | --- |
| - Age: | - 18–24 years old - 25–34 years old - 35–44 years old | - 45–54 years old - 55–64 years old - 65–74 years old | - 75 years or older |
| - Marital status: | - Single, never married - Engaged | - Married - Divorced | - Widowed |
| - Education: | - No schooling completed - Basic / compulsory education (primary – preparatory) | - High school or equivalent - Diploma (two to three years after high school) | - Bachelor’s degree - Postgraduate degree |
| - Employment status: | - Employed - Self-employed | - Student - stay-at-home | - Unemployed - Retired |
| - Country of your birth: | - Egypt - Syria | - Iraq - Palestine | - Lebanon - Other:-------------- |
| - Current immigration status | - Naturalized citizen (citizenship by conferral) - Permanent resident | - Refugee - Asylum seeker | - Other (please specify)::----------- |
| - Number of years lived in Australia: | - Less than two years - 2–4 years | - 4–6 years - 6–8 years | - More than 8 years |

- 1. Mental health and well-being state

1. Have you ever had a formal diagnosis of depression?
   - Yes
   - No
2. Have you ever received individual psychotherapy for depression (e.g., cognitive behavioural therapy CBT)?
   - Yes
   - No
3. Have you previously participated in group psychotherapy sessions for depression (for example, cognitive-behavioral therapy groups, support groups, or psychoeducational groups)?
   - Yes
   - No

**Q) How often in the last four weeks …**

| Statement/question | All of the time | Most of the time | Some of the time | A little of the time | None of the time |
| --- | --- | --- | --- | --- | --- |
| 1. Have you experienced a positive attitude towards your life? |  |  |  |  |  |
| 1. Have you felt comfortable, serene, or peaceful? |  |  |  |  |  |
| 1. Have you experienced tiredness or low energy? |  |  |  |  |  |
| 1. Have you experienced low feelings, or mood swings? |  |  |  |  |  |
| 1. Have you experienced satisfaction from what you do? |  |  |  |  |  |
| 1. Have you experienced a positive attitude towards your interpersonal relationships? |  |  |  |  |  |
| 1. Have you enjoyed spending time with family or friends? |  |  |  |  |  |
| 1. Have you felt that you’ve let yourself or others down? |  |  |  |  |  |

- 1. Cultural and Religious influence

**To complete this section, please score how strongly you agree or disagree with each of the following statements with respect to your beliefs and practices.**

| Statement | Strongly disagree | Disagree | Uncertain | Agree | Strongly agree |
| --- | --- | --- | --- | --- | --- |
| 1. Everything happens only by the will and destiny of God. |  |  |  |  |  |
| 1. Following religious practices strengthens the psychological health of the individual. |  |  |  |  |  |
| 1. Performing various acts of worship, such as prayer, and reading the Holy Book or Scripture, is a path to healing. |  |  |  |  |  |
| 1. I would prefer to turn to my religion rather than engaging with mental health treatment. |  |  |  |  |  |
| 1. Anyone experiencing mental illness should keep it hidden, even from those who are closest, to avoid the stigma. |  |  |  |  |  |
| 1. I would require family consent and support for seeking and continuing mental health therapy. |  |  |  |  |  |

- 1. Knowledge and use of existing mental health websites and applications

Q) Have you ever used the internet to look up information about mental health issues?

- Yes
- No

1. Are you aware that there are mental health websites and apps that offer psychological support and intervention?
   - Yes
   - No
2. **Have you ever tried therapeutic digital apps or online mental health programmes? *(NOTE: If your answer is No, please move to section E)***
   - Yes
   - No

Q) What is the name of the digital mental health app/program you most recently used?

**---------------------------------------------------------------------------------------------------------------------------------------**

**---------------------------------------------------------------------------------------------------------------------------------------**

Q) How long have you been using this app/program?

**---------------------------------------------------------------------------------------------------------------------------------------**

**---------------------------------------------------------------------------------------------------------------------------------------**

Q) Did you complete all of the app/program’s activities/modules? Why?

**---------------------------------------------------------------------------------------------------------------------------------------**

**---------------------------------------------------------------------------------------------------------------------------------------**

- 1. Attitude towards mHealth smartphone apps

**Please select a response following each statement corresponding to your personal view on mHealth smartphone apps.**

| Statement | Never | Rarely | Sometimes | Often | Always |
| --- | --- | --- | --- | --- | --- |
| 1. I would use an app to find information about mental health problems/concerns. |  |  |  |  |  |
| 1. I would use an app to relieve or assist me with my emotional problem/s. |  |  |  |  |  |
| 1. I would use an app to get online counselling from a psychiatrist/psychologist/psychotherapist/counsellor. |  |  |  |  |  |
| 1. I would use an app that helps me self-manage my mental health. |  |  |  |  |  |

- 1. Acceptance /Barriers to use

**What would motivate or encourage you to use a mental health app? Please respond to the following statements about what features would affect your willingness to use an app.**

| Statement | Strongly encourage use | Encourage use | Uncertain | Discourage use | Strongly discourage use |
| --- | --- | --- | --- | --- | --- |
| 1. My native language (Arabic) is an option in the app. |  |  |  |  |  |
| 1. App’s privacy policy explains how data is handled and stored. |  |  |  |  |  |
| 1. App uses data encryption measures. |  |  |  |  |  |
| 1. App enables me to get help and support while maintaining complete confidentiality of my identity. |  |  |  |  |  |
| 1. App and its content were developed with the input of specialists and health professionals. |  |  |  |  |  |
| 1. App comes from an authorized / official entity (e.g., developed by government, hospital, university). |  |  |  |  |  |
| 1. App supplies a self-assessment or management tool(s) that are medical or scientifically approved. |  |  |  |  |  |
| 1. App provides relevant and reliable information. |  |  |  |  |  |
| 1. App aids access to professional care and other mental health resources. |  |  |  |  |  |
| 1. App is free. |  |  |  |  |  |
| 1. App is simple and effortless to use (i.e., usable). |  |  |  |  |  |
| 1. Apps have customizable functionalities that allow users to tailor some app features to their needs and preferences. |  |  |  |  |  |

- 1. Useful mHealth App features

Cognitive Behavioral Therapy (CBT) is a widely-used, evidence-based form of psychotherapy that helps individuals identify and change negative thought patterns and behaviors, and is particularly effective for treating depression and anxiety disorders. It involves identifying unhelpful thoughts, challenging these thoughts, and changing behaviors to engage in positive activities, ultimately improving mood. One effective behaviour change approach in CBT to be adopted in an mHealth app is called behavioural activation, which involves engaging in positive and meaningful activities to improve mood, for example, physical exercise. Another approach is to incorporate some of the Islamic religious principles of Amal Saleh “righteous deeds” (such as acts of worship, charity and helping others) can be effective at improving well-being by motivating activity.

Mindfulness is a practice that promotes mental health by focusing on the present moment and calmly acknowledging and accepting one’s feelings and thoughts. The primary goal of this research study is to develop a mental health app that offers a self-help tool which combines mindfulness practices with principles of CBT to Arab individuals with depression but that is also grounded in cultural and religious practices**. On a scale of ‘Not at all important’ to ‘Extremely important,’ please respond to the following statements to show how important is it for you to incorporate these techniques/features/tools/contents into a mental health app that fit your needs:**

| Statement | Not at all important | Slightly important | Important | Very important | Extremely important |
| --- | --- | --- | --- | --- | --- |
| **Behavioural activation / righteous deeds / community activities**  First, thinking about Behavioral activation, it is a method used to help improve mood and reduce stress by encouraging people to engage in fulfilling or healthy activities that make them feel good. These activities can include things like nature walks, crafting, or practicing a skill.  **Now, please respond to this statement:** | | | | | |
| 1. Providing enjoyable and meaningful activities within the app to help manage stress and improve your mood. |  |  |  |  |  |
| 1. Incorporating both behavioural activation, an effective approach in CBT, a secular-based therapeutic model and some of the Islamic religious principles of ***Amal Saleh “righteous deeds”*** (such as acts of worship, charity and helping others). |  |  |  |  |  |
| 1. Incorporating activities that reflect some of the collectivistic values (such as attending family gatherings, visiting neighbours, participating in community events). |  |  |  |  |  |
| **Mindfulness/reflection/coping/relaxation**  First, thinking about Mindfulness, it is a simple practice that emphasizes being in the present moment, allowing people to pay attention to their thoughts, emotions, and sensations without judgement. For example, taking a few deep breaths while paying attention to how your body feels can help you feel more relaxed and focused.  **Now, please respond to this statement:** | | | | | |
| 1. Including mindfulness techniques within the app to help manage stress and promote emotional balance. |  |  |  |  |  |
| 1. Incorporating a feature/tool for performing ***Tasbeeh*** (i.e., a dhikr practice in Islam that involves repetitive recitation of specific phrases to praise and glorify God) to help cope with sad or distressing thoughts, emotions, and to calm down. |  |  |  |  |  |
| 1. Incorporating **Dua’a “*supplication”*** (i.e., an essential aspect of Islamic practice, referring to the act of asking Allah for help, guidance, or forgiveness. ) as a mindfulness exercise or coping tool for seeking comfort and strength can be useful. |  |  |  |  |  |
| 1. Incorporating **Tadabbur (i.e.,** a religious practice that involves observing, examining, and reflecting on the meaning of the Al-Qur’an) as a mindfulness or reflection exercise or coping tool. |  |  |  |  |  |
| 1. Incorporating **Imtinān/Shukr of Allah** “**gratitude**” as a mindfulness exercise or coping tool to help recognize and appreciate the positive aspects of the present. |  |  |  |  |  |
| **Educational/motivational content** | | | | | |
| 1. Incorporating culturally relevant ***poems*** and ***wisdom quotations*** that reflect recognizing, accepting, and replacing challenging emotions and negative thoughts with more balanced and realistic ones. |  |  |  |  |  |
| 1. Incorporating verses from the ***Quran*** and ***Hadith*** that remind one of relief after struggle, reward with patience, and that encourage acceptance, resilience, and hope. |  |  |  |  |  |
| 1. Incorporating ***narratives of prophets*** who have suffered hardship and trauma (such as Prophet Ayyub and Prophet Musa) |  |  |  |  |  |
| **User’s self-help journey** | | | | | |
| 1. **Progressive engagement path:**   A structured, step-by-step daily programme that may be used at any time of day and that gradually introduces educational content and corresponding behavioural activation and mindfulness tasks and exercises. |  |  |  |  |  |
| 1. **Gamified learning:**   Tasks and exercises introduced into the daily programme, such as challenges with rewards for completion. |  |  |  |  |  |
| 1. **Scheduled reminders:**   Allows user to schedule reminders for daily programmes at imes that suit their routines. |  |  |  |  |  |
| 1. **Contextual reminders:**   Automated reminders that alert user based on progress in completing daily programmes following periods of absence. |  |  |  |  |  |
| 1. **Self-paced learning:**   Allow user to access a supplementary educational content and engage with it in own time without being restricted by the daily programme structure. |  |  |  |  |  |
| 1. **Standalone features:**   Self-help tools (e.g., for performing tasbeeh, breathing) for coping with sad moments or distressing thoughts, accessible anytime, and includes interactive components such as a timer. |  |  |  |  |  |
| 1. **Progress tracking:**   Allows user to track progress and achieve milestones. |  |  |  |  |  |
| **Support** | | | | | |
| 1. Option to add or remove a family member or friend to engage in app to provide support and encouragement. |  |  |  |  |  |
| 1. Deliver information and educational material about related mental health concerns and issues to the selected family member or friend (supporters) in order to reduce stigma and boost their supportive capacities. |  |  |  |  |  |
| 1. Option to share specific aspects of personal progress or achievements with the selected family member or friend (supporters) (e.g., completed tasks). |  |  |  |  |  |
| 1. A messaging or commenting feature that allows selected family members or friends (supporters) to send supportive messages, comments, and feedback. |  |  |  |  |  |
| 1. Information on relevant national and local providers and mental health services for multicultural communities in Australia. |  |  |  |  |  |
| 1. Information on crisis response services that are available 24/7, and that explains how to obtain an interpreter to assist in communicating with any of those services. |  |  |  |  |  |

- **In your opinion, what, if any, are additional features/tools/contents you think can be included/adopted in smartphone-based mental health applications (apps)? (*Please write your suggestions below*)**

____________________________________________________________________________________________________________________________________________________________________________________________________________________________________________________________________________________________________________________________________________

**NOTE: This research aims to gain a better understanding of Arabs immigrants’ use, acceptance, attitudes, and opinions towards mental health apps. This will include workshops to get your opinion on the design of a prototype app. If you are interested in participating in the later stages of the study, please provide us with your contact details.**

**Name:**

**Phone Number:**

**Email:**

**Thank you for completing the survey. Your responses are valuable.**

**If you would like to be contacted for follow-up support, please provide your information below. This information will be kept confidential and used only if we are concerned for your well-being.**

**Name: __________________________________________________________**

**Phone Number: __________________________________________________**

**Email: __________________________________________________________**

**GP’s Name: _____________________________________________________**

**GP’s Phone Number: ____________________________**

## Supplemental Material S2. Arabic version of the survey

**استبيان لتقييم استخدام المهاجرين العرب تطبيقات الأجهزة المحمولة لتحسين الصحة النفسية**

يهدف هذا الاستطلاع إلى معرفة مدى استخدام مجتمعات المهاجرين العرب في أستراليا تطبيقات الأجهزة المحمولة في تحسين الصحة النفسية وكيفية استخدامها، والعوامل التي قد تؤثر على استخدامها.

قبل إجراء الاستطلاع، يرجى قراءة ملف المعلومات المرفق والموافقة على المشاركة.

شكرا لك!

**تعليمات الاستبيان:**

إن مشاركتك في الدراسة طوعية تمامًا، ولن يكون للانسحاب منها أي عواقب. ستبقى إجاباتك على الأسئلة المقدمة سرية، وستكون البيانات مجهولة المصدر لضمان عدم الكشف عن هويتك. سيتم استخدام النتائج فقط لأغراض البحث العلمي. يتضمن هذا الاستبيان عدة أسئلة حول مواضيع مختلفة. يرجى قراءة كل سؤال وجميع الاختيارات المرتبطة به قبل تحديد خيار واحد فقط من هذه الخيارات. ومن المهم ملاحظة أنه لا توجد إجابة صحيحة أو خاطئة بشكل مطلق، بل قم باختيار الإجابة الأقرب إلى أفكارك أو فهمك لكل سؤال.

قبل أن تبدأ اجابة على الاستطلاع، يرجى ملاحظة أن بعض الأسئلة قد تكون حساسة بطبيعتها. سلامتك تهمنا. إذا شعرت بضيق في أي وقت، فيرجى الاتصال بطبيبك العام أو مستشارك أو يرجى الرجوع إلى أي من الموارد المذكورة أدناه.

| **Service name** | **Description** | **Phone number** | **Web address** |
| --- | --- | --- | --- |
| Beyond Blue | يقدم خدمات مساعدة مجانية عبر الإنترنت والاتصال الهاتفي للأفراد الذين يعانون من الاكتئاب أو القلق أو مشكلات الصحة النفسية الأخرى، بالإضافة إلى أولئك الذين يسعون إلى دعم أحد أحبائهم المصابين . يمكنك الاتصال بهم 24 ساعة في اليوم، على مدار أيام الأسبوع. | 1300 22 4636 | <https://www.beyondblue.org.au/home> |
| Lifeline | يقدم دعما مجانيا للأفراد الذين يعانون من أزمة شخصية أو لديهم أفكار انتحارية. يمكنك الاتصال بهم 24 ساعة في اليوم، على مدار أيام الأسبوع | 13 11 14 | <https://www.lifeline.org.au> |
| Urgent Mental Health Care Centre (UMHCC) | خدمة مجانية، متاحة طوال اليوم وعلى مدار الأسبوع . يقدم خدمات بديلة لأقسام طوارئ مستشفى الصحة العقلية. | 0884489100 | [https://umhcc.org.au/](tel:1300%20727%20957) |
| Migrant womens services | يوفر الدعم المجاني للنساء والأطفال من دول مختلفة الغير ناطقين باللغة الإنجليزية، بما في ذلك المتحدثين باللغة العربية. كما يقدم استشارات وعلاجات للصدمات والمساعدة في الشفاء من العنف المنزلي. | ------------------ | [https://www.migrantwomensservices.com.au/domestic-violince-service/](https://www.liveslivedwell.org.au/our-services/mental-health/northern-adelaide/) |
| Survivors of Torture and Trauma Assistance and Rehabilitation Service (STTARS) | تقديم خدمات الاستشارات والدعم المجانية للاجئين والمهاجرين الذين تعرضوا للتعذيب أو الصدمة. | (08) 8206 8900 | [https://www.sttars.org.au/about/about-sttars](https://embracementalhealth.org.au/community/multilingual-information) |
| Lives Lived Well | تقديم خدمات دعم الصحة والرفاهية النفسية للعائلات والأطفال، بما في ذلك المجتمعات ذات الخلفيات المتنوعة ثقافيًا ولغويًا | [1300 727 957](https://www.beyondblue.org.au/home) | <https://www.liveslivedwell.org.au/our-services/mental-health/northern-adelaide/> |
| Embrace Multicultural Mental Health | تقديم المصادر المتاحة مترجمة إلى اللغات بما في ذلك اللغة العربية، وكذلك تقديم روابط للخدمات | ------------------ | [https://embracementalhealth.org.au/community/multilingual-information](https://www.sttars.org.au/about/about-sttars) |

**المصطلحات المهمة في الاستبيان:**

- "الصحة النفسية" تعني القدرة على التعامل مع الأزمات المتكررة، مع المحافظة على شعور إيجابي بالإنجاز والكفاية؛ ويتم تحقيقه عادة من خلال تلبية الاحتياجات الأساسية للأمن والحب، وتأكيد الذات، والإنجاز، والنجاح.

– "اضطرابات الصحة النفسية / المرض النفسي" تعني الحالات الصحية التي تنطوي على تغيرات في إدراك الشخص، أو التحكم في انفعالاته، أو مزاجه، أو تفكيره، أو تصوراته، أو سلوكه (أو مزيج منها).

- "تطبيق الصحة النفسية" هو تطبيق للهواتف الذكية موجه في المقام الأول لمساعدة المحتاجين؛ من خلال توفير أدوات لتقييم الجوانب المختلفة للصحة النفسية، والوقاية من أعراضها، ودعمها، وإدارتها.

**أ. المعلومات حول السمات والخصائص الأساسية للفرد:**

**الجنس:**

- ذكر
- أنثى

**العمر:**

- 18-24 سنة
- 25-34 سنة
- 35-44 سنة
- 45-54 سنة
- 55-64 سنة
- 65-75 سنة

**الحالة الاجتماعية:**

- أعزب، لم يسبق له أو لها الزواج
- في علاقة
- مخطوب/ أو محطوبة
- متزوج/ أو متزوجة
- مطلق/ ـو مطلقة
- أرمل/ أو أرملة

**التعليم:**

- لم يكمل/ أو لم تكمل الدراسة
- التعليم الأساسي / الإلزامي (الابتدائي - الإعدادي)
- الثانوية العامة أو ما يعادلها
- الدبلوم (من سنتين إلى ثلاث سنوات بعد المدرسة الثانوية)
- درجة البكالوريوس
- الدراسات العليا

**الحالة الوظيفية:**

- طالب
- موظف
- من العاملين لحسابهم الخاص
- رب منزل /أو ربة منزل
- عاطل عن العمل
- متقاعد

**بلد مولدك:**

- مصر
- سوريا
- العراق
- فلسطين
- لبنان
- أخرى. يرجى تحديد بلد الميلاد:

**وضع الهجرة الحالي:**

- مواطن متجنس (المواطنة عن طريق المنح)
- مقيم دائم
- لاجئ
- طالب لجوء
- أخرى. يرجى تحديد وضع الهجرة الحالي:

**عدد سنوات العيش في أستراليا:**

- أقل من عامين
- ٢-٤ سنوات
- ٥-٧ سنوات
- ٨-١٠ سنوات
- أكثر من ١٠ سنوات

**ب. حالة الصحة والرفاهية النفسية:**

س) هل سبق تشخيصك بالاكتئاب؟

o نعم

o لا

س) هل سبق لك أن تلقيت علاجًا نفسيًا لاكتئاب (على سبيل المثال، العلاج السلوكي المعرفي CBT)؟

o نعم

o لا

س) هل سبق لك أن شاركت في جلسات العلاج النفسي الجماعي للاكتئاب (على سبيل المثال، مجموعات العلاج السلوكي المعرفي، أو مجموعات الدعم، أو مجموعات التثقيف النفسي)؟

o نعم

o لا

س) يرجى الإجابة على الأسئلة التالية بناءً على عدد المرات التي حدث لك فيها مما يلي خلال الأسابيع الأربعة الماضية...

|  | أبدا | نادرا | أحيانا | غالبا | دائما |
| --- | --- | --- | --- | --- | --- |
| 1. هل تشعر بإيجابية تجاه حياتك؟ |  |  |  |  |  |
| 2. هل تشعر بالراحة، أو الهدوء، أو السلام؟ |  |  |  |  |  |
| 3. هل تعاني التعب، أو خمول في النشاط البدني؟ |  |  |  |  |  |
| 4. هل شعرت بهبوط في المشاعر أو تقلب في حالتك المزاجية؟ |  |  |  |  |  |
| 5. هل تشعر بالرضا عما تفعله؟ |  |  |  |  |  |
| 6. هل تشعر بإيجابية تجاه علاقتك الشخصية بالآخرين؟ |  |  |  |  |  |
| 7. هل تشعر بمتعة قضاء الوقت مع العائلة أو الأصدقاء؟ |  |  |  |  |  |
| 8. هل تشعر بخذلان نفسك أو خذلانك للآخرين؟ |  |  |  |  |  |

**ج. التأثير الثقافي والديني:**

لإكمال هذا القسم، يرجى تسجيل مدى موافقتك أو عدم موافقتك على كل من العبارات التالية فيما يتعلق بمعتقداتك وممارساتك

|  | لا أوافق بشدة | لا أوافق | غير متأكد | موافق | موافق بشدة |
| --- | --- | --- | --- | --- | --- |
| 1. لا يحدث شيء إلا بإرادة وقضاء الله / الرب / القوة أو القوة العليا. |  |  |  |  |  |
| 2. اتباع التعاليم الدينية تقوي الصحة النفسية للفرد. |  |  |  |  |  |
| 3. أداء العبادات كالصلاة وقراءة الكتاب والنصوص المقدسة يؤدي للشفاء النفسي. |  |  |  |  |  |
| 4. أفضّل اتباع تعاليم وأنشطة ديني من أجل الشفاء النفسي بدلاً من طلب العلاج النفسي. |  |  |  |  |  |
| 5. يجب على الشخص الذي يعاني من مرض نفسي أن يخفي ما أصابه، حتى عن أقرب الناس إليه، لتجنب العار الذي سيطاله أو يطال أي شخص عزيز عليه. |  |  |  |  |  |
| 6. أحتاج إلى مساندة أحد أفراد الأسرة ودعمه للبحث عن علاج لصحتي النفسية والاستمرار فيه. |  |  |  |  |  |

**د. مدى معرفة واستخدام المواقع والتطبيقات الإلكترونية المتاحة للصحة النفسية:**

س) هل سبق وأن استخدمت الإنترنت للبحث عن معلومات حول ما يتعلق بالصحة النفسية؟

o نعم

o لا

 س) هل لديك معرفة بوجود مواقع وتطبيقات للصحة النفسية تقدم الدعم والتدخل النفسي؟

o نعم

o لا

 س) هل سبق لك تجربة المواقع الالكترونية أو تطبيقات الهواتف الذكية المختصة بالصحة النفسية عبر الإنترنت؟

o نعم

o لا

**اذا كانت الإجابة نعم:**

س) ما اسم موقع / أو تطبيق الصحة النفسية الذي استخدمته مؤخرًا؟

س) منذ متى تستخدم هذا الموقع / أو التطبيق ؟

س) هل سبق أن أتممت جميع وحدات وأنشطة الموقع / أو التطبيق؟ ولماذا؟

**ه. الاتجاه نحو تطبيقات الهاتف الذكي في الصحة النفسية**:

يرجى تحديد إجابة لكل عبارة تتوافق مع وجهة نظرك الشخصية فيما يتعلق باستخدام تطبيقات الأجهزة المحمولة المختصة بالصحة النفسية.

|  | أبدا | نادرا | أحيانا | غالبا | دائما |
| --- | --- | --- | --- | --- | --- |
| 1. سأستخدم أحد تطبيقات الهاتف الذكي؛ للعثور على معلومات حول الصحة النفسية سواء في مشكلاتها، أو مخاوفها، أو في كل ما يتعلق بها. |  |  |  |  |  |
| 2. سأستخدم أحد تطبيقات الهاتف الذكي؛ لتساعدني في التشافي مما أواجهه من مشكلات وجدانية. |  |  |  |  |  |
| 3. سأستخدم أحد تطبيقات الهاتف الذكي للحصول على استشارة عبر الإنترنت من طبيب، أو أخصائي، أو معالج، أو مستشار نفسي. |  |  |  |  |  |
| 4. سأستخدم أحد تطبيقات الهاتف الذكي؛ التي تساعدني في الإدارة الذاتية لصحتي النفسية. |  |  |  |  |  |

**و. العوامل التي تشجعك أو تقيدك في استخدام تطبيقات الهاتف الذكي في الصحة النفسية:**

ما الذي يحفزك، أو يشجعك على استخدام تطبيقات الصحة النفسية؟ آمل منك الإجابة على العبارات التالية التي تتناول ميزات تطبيق الهاتف الذكي التي قد تؤثر على رغبتك في استخدامه.

|  | تعيق الاستخدام بشدة | تعيق الاستخدام | غير متأكد | تشجع على الاستخدام | تشجع على الاستخدام بشدة |
| --- | --- | --- | --- | --- | --- |
| 1. وجود لغتي الأم (العربية) كخيار في التطبيق |  |  |  |  |  |
| 2. توضح سياسة الخصوصية في التطبيق كيفية التعامل مع البيانات وتخزينها. |  |  |  |  |  |
| 3. يتبع التطبيق إجراءات محددة في تشفير البيانات. |  |  |  |  |  |
| 4. التطبيق الذي يتيح لي الحصول على المساعدة والدعم مع الحفاظ على السرية التامة لهويتي |  |  |  |  |  |
| 5. بُني التطبيق وما فيه من محتوى بأيدي متخصصين، ومهنيين في مجال الصحة النفسية. |  |  |  |  |  |
| 6. طُوّر التطبيق من جهة معتمدة ورسمية (على سبيل المثال، طوّر عن طريق جهة حكومية أو مستشفى أو جامعة). |  |  |  |  |  |
| 7. أدوات التقييم الذاتي، والإدارة الذاتية المتوفرة في التطبيق معتمدة طبيًا وعلميًا. |  |  |  |  |  |
| 8. يوفر التطبيق معلومات موثوقة عن الصحة النفسية. |  |  |  |  |  |
| 9. يوفر التطبيق دليل يساعد في الوصول إلى منشآت الرعاية الصحية المتخصصة، و مصادر أخرى للصحة النفسية. |  |  |  |  |  |
| 10. أن يكون التطبيق متوفرا بالمجان. |  |  |  |  |  |
| 11. سهولة استخدام التطبيق. |  |  |  |  |  |
| 12. يحتوي التطبيق على وظائف قابلة للتخصيص؛ تتيح للمستخدمين تخصيص بعض ميزات التطبيق وفقًا لاحتياجاتهم، وتفضيلاتهم. |  |  |  |  |  |

**ز. الميزات المهمة في تطبيق الصحة النفسية في الهاتف الذكي:**

العلاج السلوكي المعرفي (CBT) هو شكل من أشكال العلاج النفسي القائم على الأدلة ويستخدم على نطاق واسع؛ ليساعد الأفراد على تحديد أنماط الأفكار والسلوكيات السلبية وتغييرها، وهو فعال في علاج الاكتئاب واضطرابات القلق. ويتضمن العلاج السلوكي المعرفي تحديد الأفكار غير المفيدة، والعمل على تحدي هذه الأفكار، كما يساعد في تغيير السلوكيات للانتظام في أنشطة إيجابية؛ مما يؤدي في النهاية إلى تحسين الحالة المزاجية. إن أحد الأساليب الفعالة في العلاج السلوكي المعرفي الذي سيتم اعتماده في تطبيق الصحة النفسية في الهاتف الذكي يسمى التنشيط السلوكي والذي يتضمن الاندماج في أنشطة إيجابية وذات مغزى؛ لتحسين الحالة المزاجية على سبيل المثال: ممارسة الرياضة البدنية. ويندرج من ضمنه دمج بعض المبادئ الدينية الإسلامية كالأعمال الصالحة (مثل العبادات والصدقات ومساعدة الآخرين) هي الأخرى فعالة في تحسين الرفاهية من خلال تحفيز النشاط. وهناك أسلوب فعال آخر يعزز الصحة النفسية يسمى اليقظة الذهنية وهي ممارسة التركيز والتأمل على اللحظة الحالية، والاعتراف بمشاعر الفرد وأفكاره وقبولها.

إن الهدف الأساسي من هذه الدراسة البحثية هو تطوير تطبيق للصحة النفسية يقدم أداة مساعدة ذاتية تجمع بين ممارسات اليقظة الذهنية ومبادئ العلاج السلوكي المعرفي للأفراد العرب المصابين بالاكتئاب بالتركيز على الممارسات الثقافية والدينية. وتتضمن الدراسة مقياسا يتدرج بالاستجابات من "ليس مهمًا على الإطلاق" إلى "مهم للغاية" يرجى الرد على عباراته التالية؛ لتوضيح مدى أهمية دمج هذه التقنيات/الميزات/الأدوات/المحتويات في تطبيق للصحة النفسية يناسب احتياجاتك.

**التنشيط السلوكي/ الأعمال الصالحة/ الأنشطة المجتمعية**

إن التنشيط السلوكي طريقة تستخدم للمساعدة في تحسين الحالة المزاجية وتقليل التوتر من خلال تشجيع الأشخاص على الاندماج في أنشطة محببة وصحية تجعلهم يشعرون بالارتياح. وتشمل هذه الأنشطة مثل: المشي في الطبيعة أو الحرف اليدوية أو ممارسة إحدى المهارات.

والآن نأمل التكرم بالاستجابة على هذا العبارات التالية:

|  | غير مهم على الإطلاق | مهم قليلاً | مهم | أهميته عالية | أهميته عالية جدا |
| --- | --- | --- | --- | --- | --- |
| 1. توفير أنشطة ممتعة وهادفة داخل التطبيق للمساعدة في إدارة التوتر وتحسين حالتك المزاجية. |  |  |  |  |  |
| 1. دمج كل من التنشيط السلوكي كنموذج قائم على العلم مع بعض المبادئ الدينية الإسلامية كالأعمال الصالحة (مثل العبادات والصدقات ومساعدة الآخرين). |  |  |  |  |  |
| 1. دمج الأنشطة التي تعكس قيم الجماعة (مثل حضور التجمعات العائلية، وزيارة الجيران، والمشاركة في المناسبات المجتمعية). |  |  |  |  |  |

**اليقظة / التأمل / التأقلم / الاسترخاء**

إن اليقظة هي ممارسة بسيطة تؤكد على التواجد في اللحظة الحالية، مما يسمح للناس بالانتباه إلى أفكارهم وعواطفهم وأحاسيسهم دون إصدار أحكام عليها. على سبيل المثال، أخذ بعض الأنفاس العميقة مع إعارة الانتباه إلى ما يشعر به جسمك؛ مما يمكن أن يساعدك على الشعور بمزيد من الاسترخاء والتركيز.

والآن نأمل التكرم بالاستجابة على هذا العبارات التالية:

|  | غير مهم على الإطلاق | مهم قليلاً | مهم | أهميته عالية | أهميته عالية جدا |
| --- | --- | --- | --- | --- | --- |
| 1. تضمين تقنيات اليقظة الذهنية داخل التطبيق للمساعدة في إدارة التوتر وتعزيز التوازن العاطفي. |  |  |  |  |  |
| 1. دمج أداة أو ميزة لأداء التسبيح (أي ممارسة الذكر في الإسلام التي تتضمن تلاوة متكررة لعبارات محددة لتحميد الله وتمجيده) للمساعدة في التعامل مع الأفكار والعواطف الحزينة والمؤلمة للوصول إلى الراحة والسلام. |  |  |  |  |  |
| 1. إن دمج الدعاء (وهي جانب أساسي من الممارسة الإسلامية تشير إلى التوجه لطلب المساعدة أو المغفرة من الله) كتمرين يقظة ذهنية وأداة تأقلم للحصول على الراحة والقوة التي يمكن أن تكون مفيدة. |  |  |  |  |  |
| 1. دمج التدبّر (وهي ممارسة دينية تتضمن إدراك وتدارس وتأمل معاني القرآن) كتمرين لليقظة أو التأمل أو أداة للتأقلم. |  |  |  |  |  |
| 1. دمج "الامتنان وشكر الله" كتمرين لليقظة أو أداة تأقلم للمساعدة في التعرف على الجوانب الإيجابية للنعم الحاضرة وتقديرها. |  |  |  |  |  |

**المحتوى التعليمي/التحفيزي**

|  | غير مهم على الإطلاق | مهم قليلاً | مهم | أهميته عالية | أهميته عالية جدا |
| --- | --- | --- | --- | --- | --- |
| 1. دمج القصائد والحكم ذات الصلة ثقافيًا والتي تعكس التعرف على المشاعر الصعبة والأفكار السلبية بما يسهم في قبولها أو استبدالها بأخرى أكثر توازناً وواقعية. |  |  |  |  |  |
| 1. دمج آيات من القرآن والحديث التي تذكّر بالفرج بعد الشدة، والمكافأة بعد الصبر، وتشجع على التسليم والتعافي والأمل. |  |  |  |  |  |
| 1. دمج قصص الأنبياء الذين عانوا من الشدائد والصدمات (مثل النبي أيوب والنبي موسى) |  |  |  |  |  |

**رحلة المساعدة الذاتية للمستخدم**

|  | غير مهم على الإطلاق | مهم قليلاً | مهم | أهميته عالية | أهميته عالية جدا |
| --- | --- | --- | --- | --- | --- |
| 1. مسار التفاعل التقدمي: برنامج يومي منظم خطوة بخطوة يستخدم في أي وقت من اليوم، ويقدم بشكل تدريجي محتوى تعليميًا ومهام وتمارين في التنشيط السلوكي واليقظة الذهنية. |  |  |  |  |  |
| 1. التعلم باللعب: مجموعة المهام والتمارين التي يتم تضمينها في البرنامج اليومي، مثل التحديات مع المكافأة على إكمالها. |  |  |  |  |  |
| 1. الرسائل التذكيرية المجدولة: السماح للمستخدم بجدولة الرسائل التذكيرية للبرنامج اليومي في الأوقات التي تناسب جدوله المعتاد. |  |  |  |  |  |
| 1. الرسائل التذكيرية حسب السياق: رسائل تذكيرية آلية تنبه المستخدم لاستكمال البرامج اليومية بعد فترات الغياب بناءً على التقدم المحرز. |  |  |  |  |  |
| 1. التعلم الذاتي: السماح للمستخدم بالوصول إلى المحتوى التعليمي التكميلي والتفاعل معه في وقته الخاص دون التقيد بالبرنامج اليومي المعد. |  |  |  |  |  |
| 1. الميزات المستقلة: أدوات للمساعدة الذاتية (مثل التسبيح والتنفس) للتعامل مع اللحظات الحزينة أو الأفكار المؤلمة، يمكن الوصول إليها في أي وقت، وتتضمن مكونات تفاعلية مثل المؤقت. |  |  |  |  |  |
| 1. تتبع التقدم: السماح للمستخدم بتتبع التقدم، والأهداف المحققة. |  |  |  |  |  |

**الدعم**

|  | غير مهم على الإطلاق | مهم قليلاً | مهم | أهميته عالية | أهميته عالية جدا |
| --- | --- | --- | --- | --- | --- |
| 1. وجود خيار إضافة أو إزالة أحد أفراد العائلة أو الأصدقاء؛ للمشاركة في التطبيق لتقديم الدعم والتشجيع. |  |  |  |  |  |
| 1. تقديم المعلومات والمواد التعليمية حول مخاوف وقضايا الصحة النفسية إلى أحد أفراد الأسرة أو الأصدقاء المختارين في التطبيق كـ(داعمين)؛ لتقليل وصمة العار، وتعزيز قدراتهم الداعمة. |  |  |  |  |  |
| 1. وجود خيار لمشاركة جوانب محددة من التقدم الشخصي أو الإنجازات مع أحد أفراد العائلة أو الأصدقاء المختارين في التطبيق كـ(داعمين) (على سبيل المثال، المهام المكتملة). |  |  |  |  |  |
| 1. وجود ميزة المراسلة والتعليق تتيح لأفراد العائلة أو الأصدقاء المختارين في التطبيق كـ(داعمين)؛ لإرسال رسائل وتعليقات وملاحظات داعمة. |  |  |  |  |  |
| 1. وجود معلومات عن مقدمي خدمات الصحة النفسية الوطنية والمحلية في أستراليا للمجتمعات متعددة الثقافات. |  |  |  |  |  |
| 1. معلومات عن خدمات الاستجابة للأزمات المتوفرة على مدار الساعة طوال أيام الأسبوع، وكيفية الحصول على مترجم للمساعدة في التواصل مع أي من تلك الخدمات. |  |  |  |  |  |

**- برأيك، ما هي الخصائص أو الأدوات أو المحتويات الإضافية -إن وجدت- التي تعتقد أنه يمكن تضمنيها في تطبيقات الصحة النفسية القائمة على الهواتف الذكية؟ (يرجى كتابة اقتراحاتكم أدناه)**

**ملحوظة:**

يهدف هذا البحث الحصول على فهم أفضل لاستخدام المهاجرين العرب، وقبولهم، ومواقفهم، وآرائهم تجاه تطبيقات الصحة النفسية. ويتضمن ذلك ورش عمل للحصول على رأيك حول تصميم النموذج الأولي للتطبيق. إذا كنت مهتمًا بالمشاركة في المراحل اللاحقة من الدراسة، فيرجى تزويدنا ببيانات الاتصال الخاصة بك.

هل أنت مهتم بالمشاركة في المراحل اللاحقة من الدراسة؟

- نعم
- لا

**ملحوظة:**

حرصا منا على سلامتك وحصولك على الدعم الكافي ومن أجل التواصل معك، يرجى تقديم المعلومات الخاصة بك أدناه علما أنه سيتم الحفاظ على سرية هذه المعلومات واستخدامها فقط عند الضرورة.

هل ترغب في أن نتواصل معك؟

- نعم
- لا

## Supplemental Material S3. Correlation Coefficients and Cronbach’s Alpha of Questionnaire Categories

| Category | No. of Items | Cronbach’s Alpha | Correlation  Coefficient | Sig. |
| --- | --- | --- | --- | --- |
| Mental health and well-being state | 8 | 0.886^**^ | 0.230^**^ | 0.000 |
| Cultural and Religious influence | 6 | 0.677^**^ | 0.341^**^ | 0.000 |
| Attitude towards mHealth smartphone apps | 4 | 0.890^**^ | 0.337^**^ | 0.000 |
| Acceptance /Barriers to use | 12 | 0.945^**^ | 0.612^**^ | 0.000 |
| Useful mHealth app features | 24 | 0.948^**^ | 0.907^**^ | 0.000 |
| Total | 54 | 0.925^**^ |  |  |

^**^ Correlation is significant at the 0.01 level (2-tailed).

## Supplemental Material S4. Country of birth for ‘Other’ category in Table 3) – those born outside the regions specified in the study instrument

## Supplemental Material S5. Participant Ratings for Behavioural Activation Features

| **No.** | **Item** | **Mean** | **Percent** | **Std. Deviation** | **Agreement Level** | **Rank** |
| --- | --- | --- | --- | --- | --- | --- |
| **1** | Providing enjoyable and meaningful activities within the app to help manage stress and improve your mood. | 3.85 | 77.0 | 0.958 | Very important | 3 |
| **2** | Incorporating both behavioural activation, an effective approach in CBT, a secular-based therapeutic model and some of the Islamic religious principles of Amal Saleh “righteous deeds” (such as acts of worship, charity and helping others). | 3.91 | 78.2 | 0.996 | Very important | 2 |
| **3** | Incorporating activities that reflect some of the collectivistic values (such as attending family gatherings, visiting neighbours, participating in community events). | 3.95 | 79.0 | 0.976 | Very important | 1 |

## Supplemental Material S6. Participant Ratings for Mindfulness, Reflection, Coping, and Relaxation Features

| **No.** | **Item** | **Mean** | **Percent** | **Std. Deviation** | **Agreement Level** | **Rank** |
| --- | --- | --- | --- | --- | --- | --- |
| **1** | Including mindfulness techniques within the app to help manage stress and promote emotional balance. | 3.56 | 71.2 | 0.972 | Very important | 5 |
| **2** | Incorporating a feature/tool for performing Tasbeeh (i.e., a dhikr practice in Islam that involves repetitive recitation of specific phrases to praise and glorify God) to help cope with sad or distressing thoughts, emotions, and to calm down. | 3.76 | 75.2 | 1.169 | Very important | 4 |
| **3** | Incorporating Dua’a “supplication” (i.e., an essential aspect of Islamic practice, referring to the act of asking Allah for help, guidance, or forgiveness. ) as a mindfulness exercise or coping tool for seeking comfort and strength can be useful. | 3.90 | 78.0 | 1.131 | Very important | 2 |
| **4** | Incorporating Tadabbur (i.e., a religious practice that involves observing, examining, and reflecting on the meaning of the Al-Qur’an) as a mindfulness or reflection exercise or coping tool. | 3.90 | 78.0 | 1.165 | Very important | 2 rep. |
| **5** | Incorporating Imtinān/Shukr of Allah “gratitude” as a mindfulness exercise or coping tool to help recognize and appreciate the positive aspects of the present. | 4.14 | 82.8 | 1.077 | Very important | 1 |

## 1.7 Supplemental Material S7.Participant Ratings for Educational and Motivational Content

| **No.** | **Item** | **Mean** | **Percent** | **Std. Deviation** | **Agreement Level** | **Rank** |
| --- | --- | --- | --- | --- | --- | --- |
| **1** | Incorporating culturally relevant poems and wisdom quotations that reflect recognizing, accepting, and replacing challenging emotions and negative thoughts with more balanced and realistic ones. | 3.27 | 65.4 | 1.167 | Important | 3 |
| **2** | Incorporating verses from the Quran and Hadith that remind one of relief after struggle, reward with patience, and that encourage acceptance, resilience, and hope. | 4.16 | 83.2 | 1.068 | Very important | 1 |
| **3** | Incorporating narratives of prophets who have suffered hardship and trauma (such as Prophet Ayyub and Prophet Musa) | 4.11 | 82.2 | 1.139 | Very important | 2 |
